# Rehabilitation professionals' perspectives of factors influencing return to occupation for people with lower limb amputation in East, South, and Southeast Asian developing countries: A qualitative study

**DOI:** 10.3389/fpubh.2023.1039279

**Published:** 2023-03-02

**Authors:** Md Shapin Ibne Sayeed, Jodi Oakman, Rwth Stuckey

**Affiliations:** Centre for Ergonomics and Human Factors, School of Psychology and Public Health, La Trobe University, Melbourne, VIC, Australia

**Keywords:** occupation, rehabilitation, amputation, Asia, work

## Abstract

**Objective:**

To identify barriers and enablers for return to occupation (RTO) for people with lower limb amputation (LLA) in East, South, and Southeast Asian developing countries from the perspective of rehabilitation professionals.

**Methods:**

A convenience sample of rehabilitation professionals working in Asian developing countries participated in online in-depth interviews between September 2021 and February 2022. Interview transcripts were analyzed and thematically coded to the modified Health Care Delivery System Approach (HCDSA) framework. COREQ guidelines were followed.

**Results:**

Twenty-eight interviewees from 13 countries shared their experiences of factors related to RTO for people with LLA. Identified factors described barriers and facilitators for RTO at all four HCDSA framework levels. The “*environmental*” level had the most identified factors (*n* = 56) and the “*care team*” level the least (*n* = 31). Common environmental RTO challenges included cultural attitudes to women; lack of rural/remote services; inadequate numbers and regulation of rehabilitation professionals; inappropriate prosthesis; limited government support for rehabilitation, and reliance on charitable models.

**Conclusions:**

Despite varied cultural, religious, and geographical characteristics, consistent factors impacting RTO were identified within these thirteen countries. Identified barriers to RTO underline the need for improvements throughout service systems from the acute-care focus on saving life without consideration of RTO, the rehabilitation focused primarily on mobility, to the lack of occupational rehabilitation services and supporting policy in these countries. These interlinked factors at different levels of healthcare service systems reinforce the importance of systems approaches to best utilize limited resources toward improving RTO in this region.

## 1. Introduction

After lower limb amputation (LLA) individuals experience significant and life-long difficulties in different domains of life including occupation, requiring comprehensive interventions by multiple stakeholders (1–3). “Occupation” in rehabilitation includes self-care, productivity, and leisure, all important for good health (4). Studies from East, South, and Southeast Asian developing countries (5, 6) have identified that LLA usually impacts those in lower socio-economic groups, the young, and primary family earners, who experience difficulty returning to occupation (RTO), resulting in socio-economic stress for whole families (7–9). Despite the availability of acute care, identified gaps in this region include a lack of continuity of care between acute and rehabilitation services, as well as insufficient services and multidisciplinary professional teams to support RTO and community reintegration (7, 10). In addition to the challenges of accessing rehabilitation for LLA, most services aim to provide only basic mobility outcomes increasing challenges for return to (1, 7, 8) and retaining work (2). These challenges are increased by the lack of bio-psychosocial approaches to rehabilitation in this region, where disability considerations do not commonly address interactions between the individual, their functionality, and their environment (11). The Health Care Delivery System Approach (HCDSA) identifies important factors at four different healthcare systems levels (patient, care team, organization, and environment) to support development of initiatives to systematically target RTO after LLA (7, 12, 13).

Commonly identified barriers to RTO for people with LLA are social and workplace attitudes, support needs, gender, and rehabilitation accessibility, within the environment, home and workplace (1, 2, 14). Previous research has identified RTO barriers and enablers from the perspective of people with LLA—the service users (1, 2, 14). While users are an important information source, they have limited perspectives of the whole health care delivery system (15) and can provide only a partial understanding of this complex system (16, 17).

A multi-dimensional understanding of systems factors is necessary to underpin the design of comprehensive rehabilitation systems and evaluate barriers and enablers for RTO at all levels of healthcare (16, 17). While multiple stakeholder participation is important, this approach has been infrequently used (17). The role of rehabilitation professionals as the link between the patient and the health service delivery system, positions them as key informants who could provide a broader understanding to inform improved rehabilitation strategies (18, 19).

Exploration of barriers and enablers for RTO from the rehabilitation professional's perspective through the lens of a modified HCDSA (7) should provide valuable evidence for improving outcomes after LLA (20). Therefore, this study aimed to identify barriers and enablers for RTO for people with LLA in East, South, and Southeast Asian developing countries from the perspective of rehabilitation professionals.

## 2. Methods

### 2.1. Research question

This study asked: “What are the barriers and enablers for RTO for people with LLA in East, South, and Southeast Asian developing countries?”

### 2.2. Study design

This qualitative study used guided interviews with a convenience sample self-selected from a previously surveyed group in developing Asian countries with ethics approval from the La Trobe University - Melbourne, Australia (HEC21196) to gain insights from rehabilitation professionals about RTO for people with LLA. This study followed Consolidated Criteria for Reporting Qualitative studies (COREQ) for interviews (21).

### 2.3. Population

The participant population included rehabilitation professionals (Prosthetist and Orthotist, Physiotherapist, Occupational Therapist, Rehabilitation Counselor, Vocational trainers, etc.) working with people with LLA in the RTO stage of rehabilitation in East, South, and Southeast Asian developing countries (22). The countries of interest were selected by combining the Asian Development Bank categorization of Asian developing countries (2019) into five regions (Central Asia, East Asia, South Asia, Southeast Asia, and The Pacific) (23), and the Department of Foreign Affairs Trade (2018) list of 25 Asian developing countries (6) in the Central, East, South, and Southeast Asia regions.

### 2.4. Research team

The study was conducted by three researchers. Md Shapin Ibne Sayeed, a male with a background in physiotherapy in prosthetic rehabilitation, experience in limited-resource countries and an international rehabilitation perspective for people with amputation with previous research work in this field. Co-authors Dr. Jodi Oakman and Dr. Rwth Stuckey, are both experienced allied-health practitioners and researchers, who have worked extensively in rehabilitation, ergonomics, and human factors.

### 2.5. Recruitment

To address the lack of information about which professional function and rehabilitation roles and context in LLA rehabilitation in this region, an online anonymous survey was initially used to gather data providing insights about LLA rehabilitation in this region. Survey participants were asked questions including about service availability, funding, regulation, models and professional roles in rehabilitation service delivery in their country of practice, then invited to contact the researchers if they were interested in participating in an in-depth interview for this current study. The survey and interview studies used convenience and snowball sampling techniques for recruiting, participants were invited through various media including professional organizations (global/local) e.g., International Society for Prosthetics and Orthotics, World Physiotherapy, World Federation of Occupational Therapists, Global Cooperation on Assistive Technology; and, by email to relevant researchers identified from published journal articles. Additionally, email invitations were sent to local/international professional organizations, Non-Government Organizations (NGOs) (ICRC, HI, Exceed Worldwide, etc.), and individuals working with LLA. A professional social media page (Facebook, LinkedIn, Twitter) was created inviting participation and shared on social media pages of rehabilitation professional organizations. Snowball recruitment included inviting interview participants to share their invitation with colleagues.

Eligibility criteria were to be 18 years or older; with at least 1 year of work experience with people with LLA in East, South, and Southeast Asian developing countries; currently working. The 201 survey respondents were from 14 different countries in the region. Ninety percent were either Prosthetist and Orthotist (PO) or Physiotherapist (PT), with other represented professions: Physical Medicine and Rehabilitation doctors (2.4%), Occupational Therapists (1.9%), and others including Rehabilitation Counselors, Nurses, and support staff. The 28 qualitative study participants in this study were self-selected from the survey participants and interviewed to provide a detailed understanding of RTO service provision if they had at least 1 year of experience working as a rehabilitation professional; and were able to answer questions in English.

### 2.6. Qualitative interview

Interview participants were drawn from those who met the inclusion criteria and indicated an interest in participating in an online interview from the survey population. To maximize heterogeneity, a purposive sample was selected which aimed at representation of (when available) country, gender, and professional role. The selection aimed to include varied views by including at least 2–3 participants of each (country/sex/profession) when available. Selected participants were contacted *via* email for informed consent.

The interview schedule used semi-structured questions based on the reach, effectiveness, adoption, implementation, maintenance (RE-AIM) framework (24), and World Health Organization Template for Rehabilitation Information Collection (TRIC) (16) (see Supplementary material: Semi-structured Interview questions). Interviews were conducted between September 2021–February 2022 using Zoom or Teams online platforms and recorded. Recorded interview files were securely stored under pseudonyms. All interviews (*n* = 28) were conducted by the first author (MS); with 25 co-conducted with RS, and one with JO.

Interviews were transcribed for data analysis using the transcription platform Otter (25), carefully checked and corrected by the first author for a draft transcript. Draft transcripts were re-checked by the co- author present during each interview and provided to the interviewee for a final version. The transcripts were thematically analyzed using Braun and Clarke's (2006) proposed steps for reflexive thematic analysis (26) based on the modified HCDSA levels (7) with themes, sub-themes and key factors. *NVivo* 20 software (QSR International) was used for coding. All three authors independently coded the same 2 transcripts, then two authors independently coded the same 12 more transcripts. After comparing for consistency and validity between coders, the remaining 14 transcripts were coded using the agreed codes by one author (MS).

## 3. Results

### 3.1. Interview participant demographics

The 28 interviewees from 13 countries (see Table 1), provided over 30 hours of data. Despite extensive attempts to ensure multiple representations from all countries and professions and a heterogenous sample of the target population, no participants from some countries and rehabilitation professions were available for inclusion in the study.

**Table 1:** Interview participants demographic information (*N* = 28).

**Country of focus**	**Nationality**	**Age (years)**	**Sex**	**DAC regional country (s) of experience**	**Country of education**	**Level of education**	**Current Role**	**Work Experience (years)**	**Current organization type**	**Work location**
Afghanistan	Afghanistan	33	F	Afghanistan, Thailand	Thailand	CAT-1/BScPO	PO	-	NGO	Urban
	Afghanistan	44	M	Afghanistan	Afghanistan, India	BScPT	PT	27	NGO	Rural
Bangladesh	Bangladesh	31	M	Bangladesh	Bangladesh	BScPT	PT	4	iNGO	Rural
	Bangladesh	29	F	Bangladesh	Bangladesh	BScPT	PT	3	NGO	Urban
	Bangladesh	34	M	Bangladesh, Vietnam, Thailand	Vietnam, Thailand	CAT-1/BScPO	PO	12	NGO	Urban
	Bangladesh	33	M	Bangladesh, Vietnam	Vietnam	CAT-2/DipPO	PO	15	Private	Rural
	Bangladesh	31	M	Bangladesh, India, Thailand	India, Thailand	CAT-1/BScPO	PO	14	NGO	Rural
Bhutan	Bhutan	31	M	Bhutan, India	India	CAT-1/BScPO	PO	7	Govt	Urban
Cambodia	Cambodia	40	M	Cambodia, Sri Lanka, Indonesia, Philippines, Myanmar	Cambodia, Australia	CAT-1/BScPO	PO	23	iNGO	Rural
	Cambodia	41	M	Cambodia	Cambodia, Singapore	BScPT + MScPT	PT	15	iNGO	Urban
	Cambodia	40	F	Cambodia	Cambodia, Australia	CAT-1/BScPO + MS	V.Rehab + PO	15	Govt	Urban
India	India	48	M	India, Bangladesh,	India	CAT-1/BScPO	PO	-	iNGO	Urban
Indonesia	Indonesia	71	F	Indonesia	Indonesia	PMD + PhD	PMD	47	NGO	Rural
	Indonesia	35	M	Indonesia	Indonesia, Pakistan	CAT-2/DipPO	PO	10	Govt. Uni.Hospital	Urban
Malaysia	Pakistan	35	M	Pakistan, Malaysia	Pakistan	CAT-1/BScPO	PO	14	Private	Urban
Myanmar	Myanmar	45	M	Myanmar	Cambodia, Thailand	CAT-1/BScPO	PO	20	NGO	Rural
Nepal	Nepal	32	M	Nepal, Indonesia, Bangladesh	Nepal	BScPT	PT	12	iNGO	Urban
	Nepal	38	M	Nepal	Cambodia	CAT-2/DipPO	PO	7	Semi-Gov	Rural
	Nepal	42	M	Nepal	Thailand	CAT-1/BScPO	PO	20	Private	Urban
Pakistan	Pakistan	41	M	Pakistan	Pakistan	CAT-1/BScPO	PO	18	iNGO	Rural
	Pakistan	28	M	Pakistan	Pakistan	CAT-1/BScPO	PO	17	NGO	Rural
Sri Lanka	Sri Lanka	50	M	Sri Lanka	Sri Lanka, USA	BScPT + PhD	PT	22	Private	Urban
	Sri Lanka	40	M	Sri Lanka	Sri Lanka, Thailand	CAT-1/BScPO	PO	19	iNGO	Rural
	Sri Lanka	39	F	Sri Lanka, Thailand	Sri Lanka, Thailand	CAT-1/BScPO + MS	PO	10	Govt	Urban
Thailand	Japan	52	M	Thailand, Bangladesh, Cambodia	Japan	CAT-1/BScPO	PO	31	iNGO	Urban
	Japan	48	M	Thailand	Japan	CAT-1/BScPO	PO	27	Govt. Uni.Hospital	Urban
	Thailand	24	F	Thailand	Thailand	CAT-1/BScPO	PO	5	Govt. Uni.Hospital	Urban
Vietnam	Vietnam	25	F	Vietnam	Vietnam	BScPT	PT	3	Govt	Urban

DAC, Developing Asian Country; M, Male; F, Female; BScPT, Bachelor level physiotherapy education; MScPT, Masters level physiotherapy education; CAT-1/ BScPO, ISPO category 1 or bachelor level prosthetist and orthotist; CAT-2/ DipPO, ISPO category 2 or bachelor level prosthetist and orthotist; MS, Master of Science level education; PhD, Doctoral level education; PMD, Physical medicine doctor; PO, Prosthetist and orthotist; PT, Physiotherapist; V.Rehab, Vocational rehabilitation specialist; NGO, Non-government organization; iNGO, International non-government organization; Semi-Gov, Semi-government organization; Uni.Hospital, Hospital attached to a university; Govt., Government.

**Figure 1:**
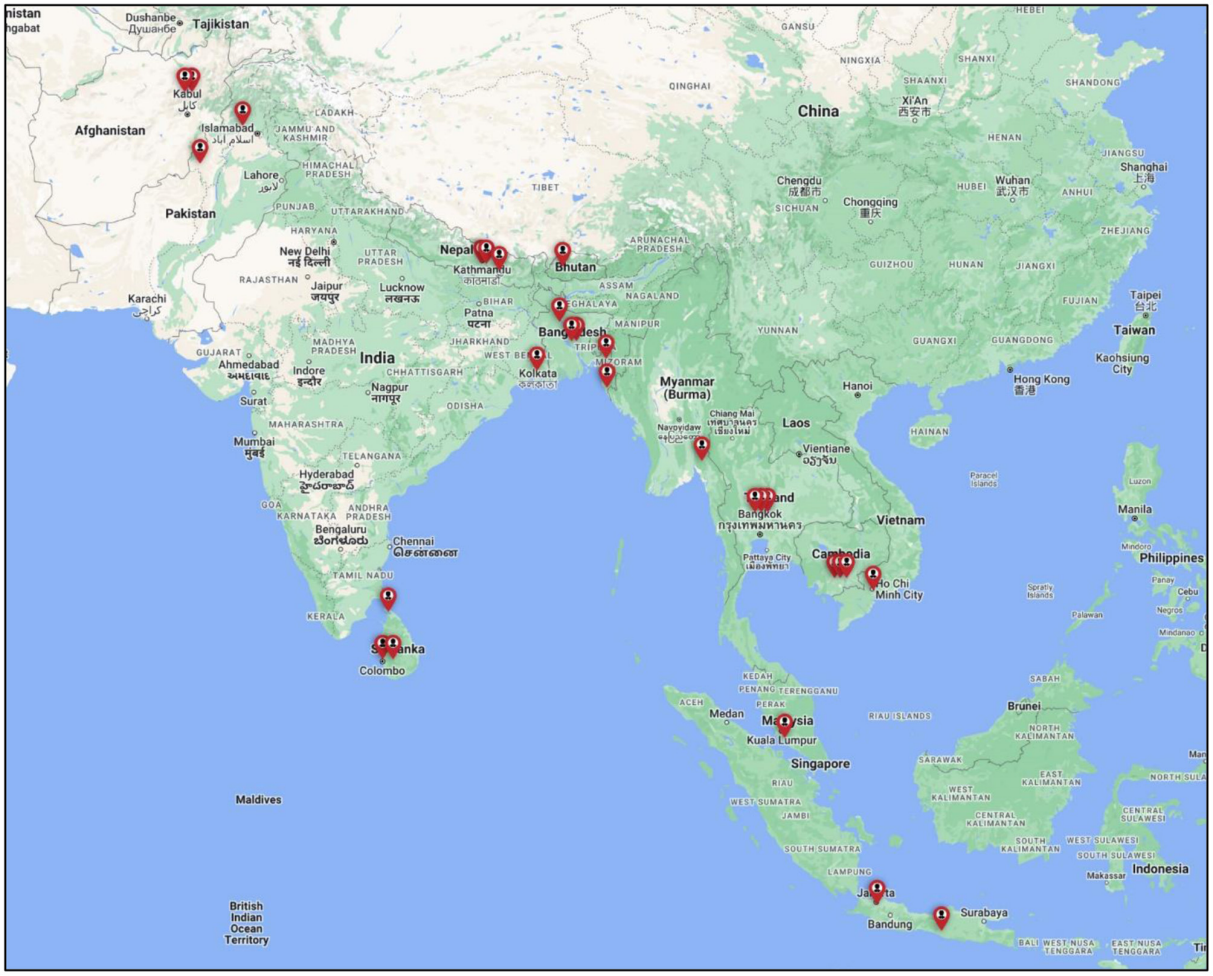
Map of participant locations. Created using Google My Maps from the following link https://www.google.com.au/maps/about/mymaps/.

All but three participants were practicing in their country of nationality. The mean age of participants was 38.57 ± 9.61 years, mostly male (*n* = 21), and almost half (*n* = 13) had experience working with LLA in multiple countries (2–5 countries). The majority (*n* = 19) had traveled to another country for their professional education. Three had diplomas, while most had bachelor or higher qualifications. Most participants were P&Os (*n* = 19) and physiotherapists (*n* = 7). One worked in vocational rehabilitation, and another was a physical medicine doctor. Fifty-seven percent currently worked in NGOs and the others in either government or semi-government organizations (29%), or private practice (14%). Participants' work experience was across 13 countries: Afghanistan, Bangladesh, Bhutan, Cambodia, India, Indonesia, Malaysia, Myanmar, Nepal, Pakistan, Sri Lanka, Thailand, and Vietnam (see Figure 1).

The interview questions enabled a deep understanding of factors related to RTO. The countries where participants worked exposed them to varied geographic, conflict, social, religious, and economic contexts, with different professional and organizational perspectives.

### 3.2. Reported socio-demographic characteristics of the LLA population

Participants were asked to describe the demographics of their country's overall LLA population (Table 2). These varied between countries and sometimes within the same country, reflecting their different locations and organizational focus (see Figure 1). Table 3 summarizes both the common and different experiences in each country. The primary cause of amputation was road traffic or industrial/work trauma. The second most common cause was pathological, commonly diabetic-related complications affecting the middle to older population. The westernization of food and lifestyle was described as a cause for increased diabetes-related cases with limited health support resulting in diabetic-related amputations. Conflict/war-related injuries leading to amputation were common for countries with current or recent conflict/war e.g., Afghanistan, and Pakistan. Both traumatic and war-related cases primarily affected the younger (18–50 years) population group.

**Table 2:** Reported socio-demographic characteristics of LLA population in each country.

**Country/number of interviews**	**Most common cause of LLA**	**Other causes of LLA**	**Description of most common age of LLA**	**Common sex of those with LLA**	**Common socio-economic status those with LLA**
Afghanistan (*n* = 2)	- Conflict/war-land mines, gunfire	- Trauma, diabetic	-Children	–	–
Bangladesh (*n* = 5)	-Trauma-RTA and industrial accidents	Pathological-diabetic and other diseases- Conflict/war- land mines/ gunfire (refugees)	-Trauma- active age (18–40 years)- Pathological- mid to old age (35–70 years)	-Male	- Lower socio-economic groups
Bhutan (*n* = 1)	-Trauma-RTA	- Pathological-diabetic and other diseases	-40–45 years	- Male	- Rural and remote area population/ farmers
Cambodia (*n* = 3)	- Trauma-RTA and industrial accident	Pathological-diabetic and other diseases- War- gunfire (refugees)	- Trauma- active age (>18 years)- Pathological- old age	Male	- Principal earners
India	Diabetic	- Trauma- RTA and industrial accidents	- Trauma- active age (>18 years)- Pathological-old age (50–60 years)	- Male	–
Indonesia	-Trauma-RTA	-Trauma-RTA	-Active age	-Male	–
	-Pathological-diabetic	-Trauma- RTA	-40–60 years	-Female	–
Malaysia	-Pathological-diabetic and other diseases	-Trauma-RTA and industrial accidents	-30–40 years	- Male	-Middle or lower socioeconomic groups
Myanmar	-Conflict/war-land mines, gunfire trauma-RTA	-Diabetic and other pathological conditions	-20–35 years	-Male	-Lower socio-economic groups
Nepal	-Trauma- RTA and natural disaster	-Trauma- Work-related accidents- Diabetic and other pathological conditions	-Active age	-Male	-Lower socio-economic groups
Pakistan	-Conflict/war-land mines, gunfire	-Pathological-diabetic trauma-RTA	−20–45 years	-Male/Female	–
	-Pathological-diabetic	-Trauma-RTA	–	–	–
Sri Lanka	-Trauma- RTA	-Pathological Diabetic and other diseases- Conflict/ war-land mines	-20–50 years	-Male	-Middle-income groups- Principal earner groups
Thailand	-Pathological-diabetic and other diseases	- Trauma-RTA and industrial accident	−30–45 years children	-Male	–
	Trauma- RTA	-Pathological-diabetic and other diseases	-Middle age (30–50)	–	–
Vietnam	-Trauma-RTA	-Pathological-diabetic and other diseases	-Trauma-active age pathological-old age (>50 years)	- Male	–

RTA, Road traffic accident; Blank spaces indicate there is no information available in the transcripts.

**Table 3:** Return to occupation for people with lower limb amputation (LLA) factors at the four levels of HCDSA, country, and relevant key outcomes—Barriers and enablers.

**HCDSA level**	**Key themes (Number of participants) Sub-themes (Number of Participants) Country (Participant no.)**	**Sub-themes Key factors of return to the occupation (RTO) ^(Participantno.)^ *Italic text- enablers;* non-italic text barriers**
Level-1: client or patient level	**Patient characteristics** (*n =* 22)	
	**Age** (*n =* 9) Afghanistan 1, 9 Cambodia 17 Nepal 20, 21 Pakistan 5, 6 Sri Lanka 22 Thailand 15	**Age** Older people experience more difficulties getting back to work 6, 9, 15, 17 Older people get less support for RTO 9 *Young people with LLA could RTO* 1, 6, 17, 22 *Children get support for education from NGOs* 1, 5, 6, 9, 20, 21
	**Sex** (*n =* 20) Afghanistan 1, 9 Bangladesh 2, 3, 4, 11, 13 Cambodia 14, 17 Indonesia 24 Myanmar 28 Nepal 20, 21, 25 Pakistan 5, 6 Sri Lanka 12, 22, 27 Thailand 18	**Sex** Females do not RTO even after rehabilitation 1, 5 Females experience additional/ unique barriers for RTO 1, 2, 3, 5, 6, 9, 11, 12, 13, 18, 22, 24, 27 Lack of support available for females to RTO 1, 2, 3, 5, 6, 9, 11, 13, 12, 20, 22 Degradation of females' role in the family/ work after LLA 3, 9, 12, 22, 25 Females with LLA have to find alternative ways to do household work 3, 4, 5, 11, 13, 28 Females are discouraged from RTO rather than being supported 6, 9, 12, 22 Females have to find alternative work that can be done from home 3, 6, 13, 14, 17, 22, 25, 27, 28 Females with a disability are considered not suitable for marriage 3, 9, 22 Females experience more shame and/or mental health issues 2, 4, 6, 9, 12, 13, 17, 18, 20, 22, 24, 27 *Females face less discrimination in the city about RTO* 14, 17, 20, 21, 24 *Male patients' RTO rate is higher* 22 *NGOs promote recruiting females with LLA* 1, 9, 11, 14
	**Economic** (*n =* 11) Afghanistan 1, 9 Bangladesh 2, 4, 11 Cambodia 17 India 7 Indonesia 24 Pakistan 5, 6 Sri Lanka 12	**Economic** Cannot afford rehabilitation and related costs- impacts RTO 4, 6, 7, 9, 11, 17, 24 Lower socio-economic groups of people with LLA experience more difficulties RTO 2, 4, 5, 6, 9, 12 The patient is the only financial source for their whole family 1
	**Patient Health Issues** (*n =* 15)	
	**Complications** (*n =* 15) Afghanistan 1, 9 Bangladesh 4, 11 Cambodia 14, 17 Myanmar 28 Nepal 20, 21 Pakistan 5, 6 Sri Lanka 12, 22 Thailand 8, 15	**Complications** Delayed access limits optimum rehabilitation outcomes and limits RTO 1, 5, 9, 20, 22 Comorbidities like diabetes make it difficult for RTO 1, 5, 8, 15 Unsuitable stump shape and size for prosthesis limits RTO 9, 21 Patients with above knee LLA experience more difficulties with RTO 1, 4, 6, 8, 11, 14, 17, 22, 28 Bilateral LLA experience more difficulties with RTO 12, 14 *Patients with below knee LLA experience fewer difficulties on RTO* 1, 4, 6, 8, 11, 14, 17, 22, 28
	**Patient Personal factors** (*n = 26)*	
	**Attitude** (*n =* 16) Afghanistan 1, 9 Bangladesh 2, 11, 13 Bhutan 26 Cambodia 14, 17 India 7 Indonesia 16, 24 Malaysia 19 Nepal 21 Pakistan 5, 6 Sri Lanka 22	**Attitude** Patients lose motivation/ self-confidence toward RTO 1, 2, 5, 6, 7, 9, 11, 13, 14, 16, 17, 21, 24, 26 Patients choose to beg or take advantage of their disability rather than work 5, 12, 11, 13, 21, 22, 26 Patient becomes dependent on family members 14, 16, 17, 21 NGOs provide everything in refugee camps so that patients need not do any work 11 Patients choose not to leave their family for vocational training 17 *Strong patient determination helps RTO* 9, 17, 19, 21, 22
	**Work Type** (*n =* 23) Afghanistan 1, 9 Bangladesh 2, 3, 4, 11, 13 Cambodia 14, 17 India 7 Indonesia 16, 24 Myanmar 28 Nepal 21, 25 Pakistan 5, 6 Sri Lanka 12, 22, 27 Thailand 8, 15 Vietnam 23	**Work Type** Physically demanding workers are downgraded to less physical work or stop work post-LLA 1, 2, 3, 4, 5, 6, 7, 8, 9, 11, 12, 13, 14, 16, 21, 22, 23, 24, 25 People working in military/ private pre-LLA experience difficulty RTO 1, 9, 16, 24, 27, 28 Principal earners of the family have to rapidly RTO 17, 22 *Government/ NGOs/ garment factories recruit and let people with LLA work* 2, 4, 5, 6, 7, 8, 12, 16, 21, 25 *Less physical demanding occupations can RTO* 3, 4, 5, 7, 8, 13, 15, 16, 24, 25
	**Literacy level** (*n =* 12) Afghanistan 1 Bangladesh 2, 3, 4, 11 Cambodia 10, 14 Indonesia 24 Nepal 21 Pakistan 5 Sri Lanka 12, 22	**Literacy level** People with LLA with low-levels of education experience difficulty RTO 1, 2, 3, 4, 5, 10, 11, 12, 14, 21, 22, 24
	**Information** (*n =* 17) Afghanistan 9 Bangladesh 2, 3, 4 Bhutan 26 Cambodia 10, 14, 17 India 7 Indonesia 24 Malaysia 19 Nepal 21, 25 Pakistan 5, 6 Sri Lanka 12, 22	**Information** People have limited awareness of rehabilitation and RTO opportunities 2, 3, 6, 7, 9, 10, 12, 14, 21, 22, 24, 25 *Social media/ awareness programs are helping people to RTO* 2, 4, 5, 6, 10, 12, 17, 19, 21, 26
**Level-2: Care Team**	**Family and Friends** (*n =* 21)	
	**Peer support** (*n =* 14) Afghanistan 1 Bangladesh 2, 3, 11 Bhutan 26 India 7 Nepal 20, 21, 25 Pakistan 5 Sri Lanka 12, 22 Thailand 8, 18	**Peer support** No structured peer support groups available 1, 2, 3, 5, 7, 8, 11, 18, 20, 21, 22, 25, 26 *Rehabilitation staff with disability set examples* 1, 2, 3, 12 *Occasional organizational programs provide some form of peer support* 5, 7, 20
	**Family support** (*n =* 16) Afghanistan 1, 9 Bangladesh 2, 3, 4, 11 Cambodia 14, 17 India 7 Indonesia 16 Nepal 21 Pakistan 6 Sri Lanka 12, 22 Thailand 18 Vietnam 23	**Family support** Lack of family support for rehabilitation limits RTO 1, 6, 9, 11, 12, 14, 16, 17, 18 Lack of support from family for RTO 3, 4, 9, 17, 21, 22, 23 Difficulties adjusting to the suddenly changed roles within the family 1, 2, 3 Family is dependent on the people with LLA 1, 2, 17 *Family members support RTO or help financially to start a business* 2, 7, 23
	**Acute care Team** (*n =* 17)	
	**Referral** (*n =* 14) Afghanistan 1 Bangladesh 2, 3, 4, 11, 13 Cambodia 10, 14 Malaysia 19 Myanmar 28 Nepal 25 Pakistan 5, 6 Sri Lanka 22	**Referral** Lack of referral for rehabilitation and/ or vocational training limits RTO 1, 2, 3, 4, 5, 6, 10, 11, 13, 14, 19, 22, 25, 28
	**Surgical considerations** (*n =* 7) Afghanistan 1, 9 Bangladesh 11 Bhutan 26 Malaysia 19 Myanmar 28 Thailand 18	**Surgical considerations** Surgeons not considering RTO plan for ideal stump for a prosthesis 1, 9, 11, 18, 19, 26, 28
	**Rehabilitation Team** (*n =* 22)	
	**Professional challenges** (*n =* 10) Afghanistan 1, 9 Bangladesh 4 Cambodia 14 India 7 Myanmar 28 Nepal 21, 25 Sri Lanka 22 Thailand 18	**Professional challenges** Lack of professionals working for RTO in the country 1, 9, 14, 18, 21, 22, 25 Professionals working for RTO do not get opportunity to be involved in rehabilitation 1, 7, 9, 14, 18, 21, 22, 25, 28 The workload for available professionals' limits outcome 7 Technological limitations create a negative image of the professions 4, 7
	**Gender in profession** (*n =* 16) Afghanistan 9 Bangladesh 3, 4, 11, 13 Cambodia 10, 14, 17 India 7 Indonesia 16 Nepal 25 Pakistan 5, 6 Sri Lanka 12, 22, 27	**Gender in profession** Less female professionals' limits female patients rehabilitation and RTO 3, 4, 5, 6, 7, 9, 10, 11, 12, 13, 14, 16, 17, 22, 25, 27
	**Multi-disciplinary Team** (*n =* 17) Afghanistan 1, 9 Bangladesh 2, 3, 11, 13 Cambodia 10, 14 India 7 Myanmar 28 Nepal 21, 25 Pakistan 5 Sri Lanka 12, 22 Thailand 8, 18	**Multi-disciplinary Team** The lack of team professionals important for RTO 1, 3, 7, 9, 13, 14, 18, 21, 22, 25, 28 Rehabilitation aims focus on mobility and not RTO opportunities 1, 2, 5, 7, 8, 10, 11 *MDT works together to plan and support ADL/ home duties* 12
**Level-3: Organization level**	**Service Facility** (*n =* 26)	
	**Availability and location** (*n =* 22) Afghanistan 1, 9 Bangladesh 2, 3, 4, 11, 13 Bhutan 26 Cambodia 14, 17 Indonesia 16, 24 Myanmar 28 Nepal 20, 21 Pakistan 5 Sri Lanka 12, 22 Thailand 8, 15, 18 Vietnam 23	**Availability and location** RTO service centers unavailable or very limited and in city areas 1, 3, 4, 5, 9, 11, 13, 17, 18, 20, 21, 22, 23, 28 Available vocational rehabilitation center has very limited capacity compared to need 2, 4, 5, 9, 11, 12, 14, 16, 17, 20, 23, 26 Services targeted to the people close to the center for monitoring 1 *Vocational training available if patients need them* 2, 4, 8, 12, 14, 15, 16, 24, 26
	**Support** (*n =* 25) Afghanistan 1, 9 Bangladesh 2, 3, 4, 11, 13 Bhutan 26 Cambodia 10, 14, 17 India 7 Indonesia 16, 24 Myanmar 28 Nepal 20, 21, 25 Pakistan 5, 6 Sri Lanka 12, 22 Thailand 8, 18 Vietnam 23	**Support** Lack of support for RTO 1, 2, 3, 4, 5, 6, 7, 8, 9, 11, 13, 18, 20, 21, 22, 23, 24, 25, 26 No support for hi-tech components if required for RTO 1, 2, 3, 4, 6, 7, 9, 11, 18, 23, 26 Lack of financial support for starting their own business 4, 5, 9, 12, 18, 22
		Lack of support from the workplace for people with LLA to RTO 1, 2, 3, 14, 17, 23, 24, 25, 26 Lack of support to produce prosthetic components locally/ meeting the contextual need 7 No mental health support 7 No insurance or Workcover available 4, 22 Inequality in services 3 *Organizations providing training for improving mobility and ADL at home* 2, 6, 12, 17, 20, 25, 26, 28 *Staff with disability working in rehabilitation centers could motivate RTO 1 NGOs supporting job opportunities and skill improvement training* 1, 4, 8, 9, 10, 11, 13, 17 *CBR program helping people RTO* 16, 21 *Some NGOs are providing financial support to start a small business* 4, 13, 25
	**Service delivery** (*n =* 28)	
	**Service model** (*n =* 20) Afghanistan 1, 9 Bangladesh 2, 3, 4, 11, 13 Cambodia 10, 17 India 7 Indonesia 16, 24 Myanmar 28 Nepal 20, 21 Pakistan 5 Sri Lanka 12 Thailand 8, 15 Vietnam 23	**Service model** NGOs provide support based on fund availability 20 *NGOs providing RTO service in the country* 1, 2, 3, 4, 5, 7, 9, 10, 11, 13, 20, 21, 23, 28 *Government providing vocational training for disabled people* 7, 8, 12, 15, 16, 17, 24
	**Service quality** (*n =* 23) Afghanistan 1, 9 Bangladesh 2, 3, 4, 11, 13 Bhutan 26 Cambodia 10, 14, 17 India 7 Indonesia 16 Malaysia 19 Nepal 20, 21 Pakistan 5, 6 Sri Lanka 12, 22 Thailand 8, 18 Vietnam 23	**Service quality** Lack of technology to provide customized services to promote RTO 1, 2, 3, 4, 5, 6, 7, 8, 9, 11, 12, 13, 14, 16, 18, 19, 20, 21, 23, 26 The prosthesis is good for life-activities but not for work 12, 19, 23 Government vocational training quality needs to be improved 7, 9, 10, 11, 12, 13, 14, 17, 22 Government vocational training options are not based on the demands of job sectors 7, 14, 17 COVID-19 impacting vocational training center access 17
	**Service resources** (*n =* 15) Afghanistan 1, 9 Bangladesh 2, 3 Bhutan 26 Cambodia 10, 14, 17 Indonesia 24 Nepal 21, 25 Pakistan 5, 6 Thailand 18 Vietnam 23	**Service resources** Lack of funds supporting the need to support RTO 1, 6, 18, 21 Lack of workplace physical accessibility for people with disability 1, 2, 3, 5, 6, 9, 14, 17, 18, 23, 24, 25, 26 Developed and using foot supporting contextual work/ cultural requirements 10, 14
	**Service strategy** (*n =* 25) Afghanistan 1, 9 Bangladesh 2, 3, 4, 11, 13 Bhutan 26 Cambodia 10, 14, 17 India 7 Indonesia 16 Myanmar 28 Nepal 20, 21, 25 Pakistan 5, 6 Sri Lanka 12, 22, 27 Thailand 8, 18 Vietnam 23	**Service strategy** RTO is neglected while considering rehabilitation plan 7, 18 Prosthesis requires wearing shoes which is not work/ culturally acceptable in the region 1, 2, 4, 6, 7, 8, 9, 10, 11, 12, 13, 14, 18, 20, 21, 22, 23, 26, 28 Lack of workplace familiarity of the ability of people with LLA and unwillingness to retain their jobs 1, 2, 22, 23 *Training program for health professionals about RTO service 12 NGOs support home modification to assist ADL 11, 17 Organizations changing strategies to recruit people with disability* 2, 3, 4, 5, 6, 8, 12, 16, 18, 21, 25 *Prosthetic foot can support farming* 10, 14 *People with LLA from the military are playing in national/ international disability sports* 27
**Level-4: Environment level**	**Economic** (*n =* 27)	
	**Cost** (*n =* 26) Afghanistan 1, 9 Bangladesh 2, 3, 4, 11, 13 Bhutan 26 Cambodia 10, 14, 17 India 7 Indonesia 16, 24 Malaysia 19 Myanmar 28 Nepal 21, 25 Pakistan 5, 6 Sri Lanka 12 Thailand 8, 18 Vietnam 23	**Cost** Difficulty affording rehabilitation limits RTO 1, 2, 3, 4, 5, 6, 7, 8, 9, 10, 11, 12, 13, 14, 16, 18, 21, 22, 23, 24, 25, 27, 28 Difficulty paying for the hi-tech prosthesis which can support work-role functionality 1, 2, 3, 4, 5, 6, 7, 9, 10, 12, 13, 14, 18, 19, 21, 23, 25, 26, 27 *Vocational training is free* 2, 3, 9, 10, 11, 12, 14, 17, 24, 26
	**Financial support** (*n =* 25) Afghanistan 1, 9 Bangladesh 2, 3, 4, 11, 13 Bhutan 26 Cambodia 10, 14, 17 India 7 Indonesia 16, 24 Malaysia 19 Myanmar 28 Nepal 20, 21 Pakistan 5, 6 Sri Lanka 12, 27 Thailand 8, 18 Vietnam 23	**Financial support** Difficulty paying for rehabilitation limits RTO 2, 3, 4, 5, 6, 7, 8, 9, 13, 16, 18, 19, 20, 21, 23, 24, 27 Lack of financial support for starting a small business 2, 5, 6, 7, 11, 13, 17, 20, 21, 26, 28 No lack of government RTO support services 1, 3, 5, 6, 9, 11, 13, 20, 23, 26, 28 *Some NGOs financially support setting up a small business* 1, 6, 10, 14, 17, 20, 26 *The government has a scheme to provide small monthly financial support* 3, 7, 12, 21, 27, 28
	**Physical and Social Environment** (*n =* 28)	
	**Societal and workplace attitudes** (*n =* 24) Afghanistan 1, 9 Bangladesh 2, 3, 4, 11, 13 Cambodia 10, 14, 17 India 7 Indonesia 16, 24 Myanmar 28 Nepal 20, 21, 25 Pakistan 5, 6 Sri Lanka 12, 22 Thailand 8, 18 Vietnam 23	**Societal and workplace attitudes** Negative attitudes from society and workplace toward RTO 1, 2, 3, 4, 5, 6, 9, 10, 11, 12, 13, 14, 16, 17, 18, 20, 21, 22, 23, 24, 25, 28 Family or colleagues' sympathetic behavior discourages RTO 5, 6, 7, 9, 12, 17, 18, 22, 25 In remote areas people with a disability are not allowed to go to school/work 1, 9 *Positive attitude from city/educated people and workplaces toward RTO* 8, 10, 11, 12, 20, 21, 28
	**Home Environment and ADL** (*n =* 17) Bangladesh 2, 3, 11 Bhutan 26 Cambodia 14, 17 Myanmar 28 Nepal 20, 21, 25 Pakistan 6 Sri Lanka 12, 22, 27 Thailand 8, 15, 18	**Home environment and ADL** Lack of home environment modification creating mobility and ADL difficulties 3, 8, 11, 14, 15, 17, 21, 22, 25, 26, 27, 28 *Some modification to home environments to improve ADL* 2, 6, 12, 17, 20, 25, 26, 28 *Most home environments in the city areas do not limit mobility and ADL* 3, 15, 17, 18, 20, 21, 27
	**Transport and terrain access** (*n =* 20) Afghanistan 1, 9 Bangladesh 11, 13 Bhutan 26 Cambodia 10, 14, 17 Indonesia 16, 24 Malaysia 19 Myanmar 28 Nepal 21, 25 Pakistan 5 Sri Lanka 12, 27 Thailand 8, 15 Vietnam 23	**Transport and terrain access** Roads and transport availability/system makes it difficult for people with LLA to RTO 1, 6, 9, 10, 11, 12, 13, 14, 16, 17, 21, 23, 24, 25, 26, 27, 28 Mountainous terrain creates mobility difficulties for people with LLA for RTO 1, 6, 9, 10, 13, 16, 17, 23, 24, 25, 26, 27, 28 Environmental factors create mobility difficulties for people with LLA for RTO 1, 6, 16, 17, 21, 28 *Roads and transport availability/systems for people with LLA support RTO* 8, 15, 19
	**Political Factors** (*n =* 26)	
	**Conflict and security issues** (*n =* 13) Afghanistan 1, 9 Bangladesh 11 Cambodia 10, 14, 17 Myanmar 28 Nepal 25 Pakistan 5, 6 Sri Lanka 12, 22 Vietnam 23	**Conflict and security issues** Long-term conflict/ war impacts countries rehabilitation, work availability and RTO 1, 5, 6, 9, 10, 12, 14, 17, 22, 23, 25 Increasing numbers of patients and RTO service requirements due to conflict/ war 1, 5, 6, 9, 28 Conflict/war causes people to experience economic hardship 9, 28 Refugees do not get work opportunities in their camp 11
	**Policy** (*n =* 22) Afghanistan 1, 9 Bangladesh 2, 3, 11, 13 Bhutan 26 Cambodia 10, 14 India 7 Indonesia 16 Malaysia 19 Myanmar 28 Nepal 20, 25 Pakistan 5, 6 Sri Lanka 12, 22, 27 Thailand 15 Vietnam 23	**Policy** No/weak government RTO support policies for people with LLA 1, 2, 5, 6, 9, 11, 13, 14, 22, 23, 25 No/lack of rehabilitation and RTO services for LLA in health policy 1, 3, 5, 9, 10, 11, 13, 14 No/lack of policy implementation in the country 2, 3, 13, 14, 16, 19, 25, 28 Long bureaucratic systems to access support services 5, 7, 15, 25, 27 NGO's interest in a specific group/area limits service to others 5, 11 Lack of government awareness about rehabilitation 6, 22 Dependent on fund availability from donors 20 *Government initiating health support for rehabilitation and re-integration* 3, 5, 12, 13 *Government inclusion of policy for rehabilitation for LLA is supporting people* 12, 26 *NGOs support children's education and supply study material* 6
	**Country service model** (*n =* 18) Afghanistan 1 Bangladesh 2, 3, 4, 11, 13 Bhutan 26 Cambodia 10, 14 India 7 Myanmar 28 Nepal 25 Pakistan 5, 6 Sri Lanka 22, 27 Thailand 8 Vietnam 23	**Country service model** Lack/weak government RTO service model 1, 2, 3, 4, 5, 6, 7, 11, 22, 23, 25, 26, 27, 28 Rehabilitation separates from the mainstream health system 10, 14 Charitable support models used for political outcomes 7, 8, 10, 13 *Organizations for disabled people advocating governments to improve RTO* 10
	**Targeted service provision** (*n =* 10) Afghanistan 1 Bangladesh 11, 13 Cambodia 10, 17 India 7 Indonesia 24 Myanmar 28 Nepal 25 Pakistan ^6^	**Targeted service provision** Lack of information about the LLA population characteristics and RTO ^1, 7, 10, 17, 25, 28^ Lack of targeted awareness programs and seminars for health professionals ^6, 13^ *Awareness programs improving RTO* ^11, 13, 24^

LLA, Lower Limb Amputation; NGOs, Non-government Organization; MDT, Multi-disciplinary Team; ADL, Activities of Daily Living; CBR, Community Based Rehabilitation; COVID-19, Coronavirus Disease of 2019.

### 3.3. Reported rehabilitation and RTO setting

Participants described rehabilitation RTO services for LLA as primarily dependent on NGOs in these countries because of the lack of inclusion of rehabilitation in core health service programs. Whilst some countries provide government rehabilitation services and a few RTO including vocational services, these are insufficient to match demand. Despite the provision of well-established acute care services, their lack of integration with rehabilitation and community care is a significant gap, compounded by the focus on physical rehabilitation, mobility and prosthetic provision with little consideration of vocational, social and community functions and demands. This was described as symptomatic of the limited professional skills available to form MDTs and supplement the skills of the available PO and PT.

### 3.4. Themes and sub-themes

**Figure 2:**
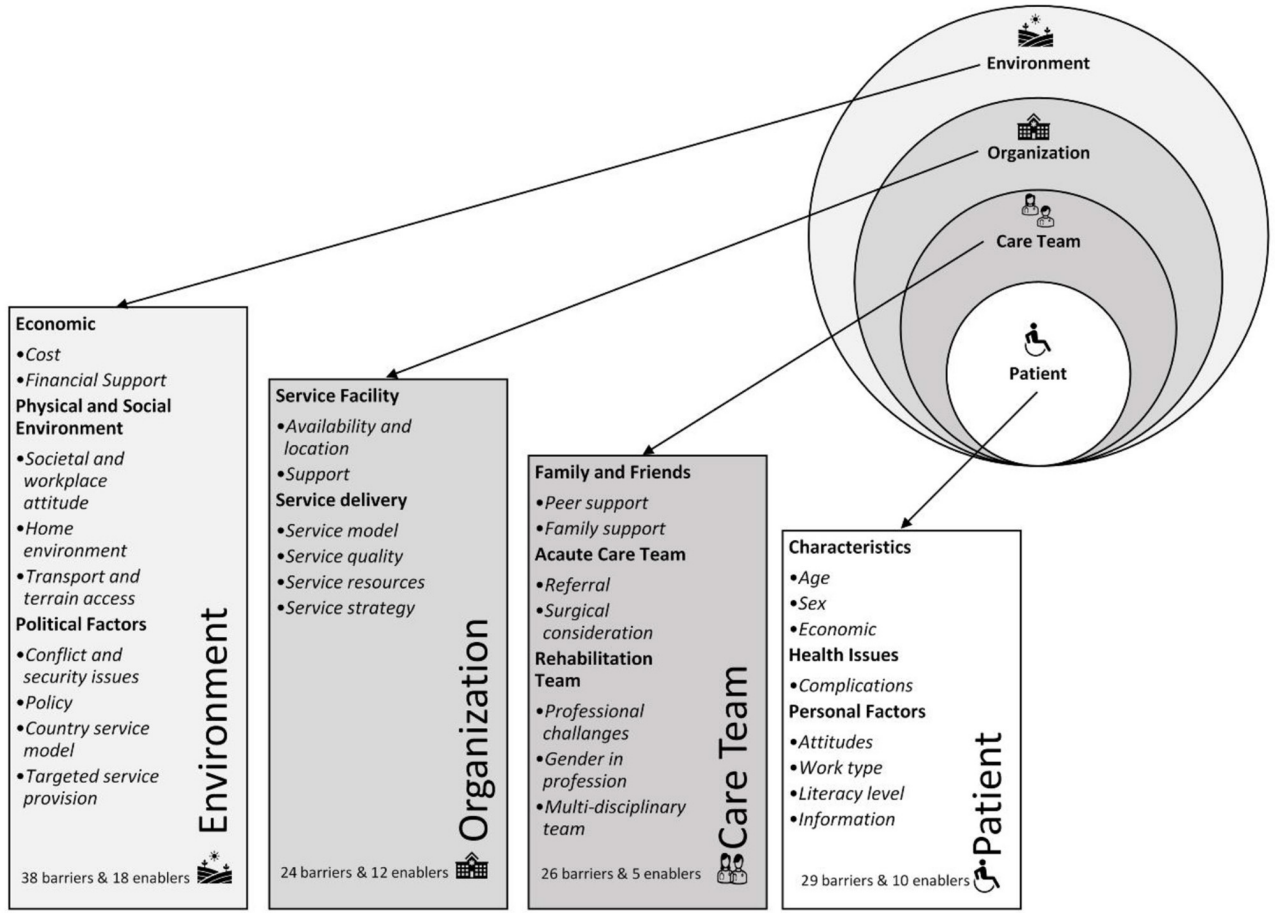
Conceptual framework with themes and sub-themes. Adapted from Ferlie and Shortell (27).

Eleven study themes and 31 sub-themes were identified describing factors related to barriers and facilitators for RTO. These were organized within the four levels of the HCDSA framework (see Figure 2).

This four-level framework proposed by Ferlie and Shortell (27) explains the healthcare delivery system model and the structural dynamics between patients, the care team, the organization, and the environment (27). The “*patient”* at the center, is surrounded by the “*care team*” including professionals and family members; then the system or “*organization*”, its service delivery, resources, and strategies; with the outer level being the “*environment*” including political, economic and physical structures (12). The “*environmental*” level had the most identified factors (*n* = 56) while the “*care team*” level had the least (*n* = 31). All factors identified at each HCDSA level for each theme and sub-themes are presented in detail in Table 3, with the relevant country/s identification for each theme. This section presents a summary of the most frequently identified factors, themes and sub-themes across these countries.

#### 3.4.1. Level-1: Patient factors

‘*Age*' impacts RTO as older people struggle more than younger to RTO (four countries).

“*Young can take their responsibilities and go back to a normal life. But getting back to work is not as successful for elder people as they don't get the facility, so this is tough that they cannot go back to work”* (Afghanistan).

“*Sex”* was significant in RTO (9 countries), especially for females who experience unique additional barriers (6 countries) compared to men, with less support for RTO (five countries), and often needing to find alternative work which can be undertaken from home (six countries).

“*After training, if they can't go to paid employment, at least they will do their shop, grocery shop or something, or maybe they have one machine to sew”* (Cambodia).

Culturally women with LLA were reported to experience higher levels of mental ill-health (seven countries) as they had to modify their previous household work-roles (three countries) and accept demeaning positions at their home or workplace (four countries). Some were also now considered unsuitable for marriage (three countries).

“*The men, they don't feel much different, but the females feel different because they cannot get married. Because they don't get considered for marriage”* (Sri Lanka).

However, participants from Cambodia, Malaysia, and Nepal reported females face less discrimination in city areas, and organizations are working to promote female patients with LLAs' RTO.

“*I don't know about the remote areas, in the villages, but in the city areas, they don't have any discrimination and they easily get back to their work”* (Nepal).

“*Economic”* factors are important with amputation most often impacting lower socio-economic groups which increases RTO difficulty (four countries), as rehabilitation or related costs impose further barriers (six countries).

“*They're not well-educated and are from labor-intensive jobs. They get it difficult to get back to the work”* (Bangladesh).

Comorbidities (three countries), unsuitable stump shape/size (two countries), and complications due to delayed access to rehabilitation (four countries) created further RTO difficulties.

“*Diabetic patient with prosthesis has a higher risk of blisters, risk of further complications of the stamp. So, they must be careful. For most of them it is a challenge to reintegrate”* (Afghanistan).

“A*ttitude*” (10 countries) includes participants noting that many develop dependency on their family (three countries) while Young and active people were described as losing their self-confidence to RTO, even with rehabilitation support (eight countries).

*Hard-working people, breadwinners usually come back very fast. They don't have any support. So, they have to work hard, even some don't come for rehab, but they go to field, and start working with one leg”* (Sri Lanka).

Interestingly some patients chose to beg, taking advantage of their disability rather than finding work (five countries).

“*Some people use their disability for income, like they beg with the prosthesis, they show the prosthesis or the disability.”* (Sri Lanka).

Previous “*work type*” is important, those previously in physically demanding work are usually downgraded to light work or stop working (nine countries).

“*if the person was from desk or official, mostly they can return. But if they need to run, walk in previous job, self-employed and entrepreneur, or salary in store, they have trouble”* (Indonesia).

Patients with lower ‘*literacy levels*' usually experienced greater challenges than those with higher literacy (seven countries).

“*If they are working, so definitely they are educated people. If they are not, then it is difficult for RTO”* (Pakistan).

Lack of “information” about RTO service availability impacts skill development opportunities (nine countries), while social media was identified as raising service awareness and improving both patient and community attitudes about RTO (sox countries).

“*Promotion, awareness material and awareness through the social media helped people to understood, and they see the ability of people, rather than stigma or having some degree of discrimination toward person with disability”* (Cambodia).

#### 3.4.2. Level-2: The care team

The availability of peer-group support for RTO was limited with three countries having occasional peer-disability programs and another three describing deliberate recruitment of people with a disability as role models.

“*We don't have this here yet, but with physiotherapist, when we see these patients then we try to make appointments for them to meet each other like that. But officially we don't have”* (Thailand).

*Family support*' for rehabilitation participation and then RTO was identified as important in 10 countries while the lack of support for females or children identified as impacting their RTO potential (seven countries).

“*Her husband is working abroad, and son is a full-time banker. So, she was not able to get any help or motivation from the family”* (Nepal).

Lack of “*Referral”* after acute care was identified as a serious challenge for both rehabilitation and RTO (seven countries).

“*So, some people after amputation go home without knowing about rehabilitation services”* (Sri Lanka).

“S*urgical considerations*” by acute care teams impact RTO as non-ideal stump shape, size, or suture lines compromise prosthetic fitting and patient functionality.

“*I have seen like the bones are very prominent. They didn't cover the bones properly, sharp bones, neuroma and then the immobile scar, scars on their patella tendon area, difficult for prosthetic fitting”* (Malaysia).

“*Professional challenges*” particularly, the lack of rehabilitation professions/professionals impact RTO services with lack of RTO skills in the rehabilitation teams (7 countries).

“*We do not have occupational therapists. This (RTO) is covered by physiotherapists, which is not enough, because to reintegrate to their previous job, all aspects should be considered”* (Afghanistan).

The impact of the lack of female professionals extends to RTO in these regions where many females are culturally shielded, resulting in difficulties for their RTO including doing household activities (eight countries).

“*Gender is not balanced across the country, some center they have 2–3 female professional, but some have only male professional. Also, female professionals don't want to work in the regional/ provincial areas. So, they (patients) have no choice”* (Cambodia).

The need for the rehabilitation team to include a range of professionals Is a challenge with most reporting teams of only physiotherapists and P&O, lacking those with important skills for RTO e.g., occupational therapist, vocational trainer etc. (eight countries), and without a focus on RTO.

“*When they go for rehabilitation, I have doubt how many prosthetists or how many physiotherapists ask them, what is your job? what you want to do after this?”* (India).

#### 3.4.3. Level-3: Organization

In some locations vocational training centers are available (six countries) but with limited capacity (eight countries).

“*From the ministry of health there are some centers for training. But I think only very few people with amputation go to the center. Only the one who is fortunate. But many of them is still outside because there is limited capacity”* (Indonesia).

Some improvements described to increase ‘s*upport*' at the organizational level and help improve developing skills for RTO (4 countries) were community-based approaches (two countries) or small funded projects (2 countries). However, for most a lack of support within their rehabilitation program for RTO was a concern (10 countries).

“*Our services are not specialized for return to work, we are trying to make them functional, that is the main priority, because there are different kinds of support they need, this is not really meeting their ideal needs”* (Nepal).

Most organizations provide basic functional prostheses, unsuitable for patients requiring hi-tech devices to undertake physically demanding occupations (seven countries). Many workplaces were not supportive of RTO (seven countries), with patients who wished to start a business also experiencing difficulties due to lack of funding (four countries).

“*The design of the workplace is not helping to go back to work at the same place as after amputation, because they used to be working laboriously in the factory to bring food to family, but now they just cannot go back to work in that area”* (Cambodia).

RTO services for LLA are limited with two common ‘*service model(s)*', services provided through NGOs (eight countries) and/or government (five countries).

“*Some NGOs do the reintegration program. Like, job training plus giving some cash, so that they can start some business and for people who have business”* (India).

“*Service quality*” again included prosthetic technological limitations (11 countries), with currently available basic prostheses.

“*We have huge technological limitations. For example, my service user needs to walk around 10 kilometers for their work, but I'm giving the prosthesis, which is maybe for two kilometers, that never meets the need”* (Bangladesh).

The quality of government vocational training centers (five countries) including the mismatch between trades-skills offered and jobs available, limits RTO success for those who can access training.

“*The vocational training does not match jobs available in the country. Same people get trained for five/ six skills and come back again”* (Cambodia).

“S*ervice resources*” impact service capability and thus RTO. Significant concerns were lack of workplace physical access for people with a disability (9 countries) and funds to support RTO (four countries). “S*ervice strategy*” issues include factors such as provision of prosthetic feet which require shoes when this is not the work/cultural norm (10 countries).

“*Normally, prosthetic foot is designed to use with footwear. But mostly they use without footwear and come back with broken foot”* (Sri Lanka).

The lack of employers' understanding of ability of people after LLA, or willingness to retain their employment (four countries) is a challenge, however, some government and NGO organization are introducing strategies to improve RTO support, starting from modifying homes (six countries).

“*Community workers go to assess their home environment and then provide accessible toilet. Sometimes, we help them to make toilet that is suitable for a person with prosthesis.”* (Cambodia).

#### 3.4.4. Level-4: Environment

“*Cost*” negatively impacts appropriate prosthetic provision and RTO in all countries except Bhutan and Malaysia, while free vocational training is a positive RTO support (six countries).

“*For the national vocational training, they don't have to pay. It's free of charge, and there is also accommodation for them.”* (Cambodia).

“*Financial support*” includes the lack of support for patients' rehabilitation costs, impacting their RTO potential (10 countries), with some modest financial support available through government' disability support schemes (five countries), while for others no government RTO support is available (six countries).

“*Nowadays government give some monthly pay to the disabled people including amputation people, maybe $3 to $4 per month”* (Bangladesh).

“S*ocietal and workplace attitudes*” include negative community and/or workplace attitudes, a major barrier for RTO (10 countries). Additionally, overprotective behavior by family members or colleagues' can be a RTO disincentive.

“*If they do not perform their activities independently in the workplace, as a patient they are already depressed and then getting too much attention makes it worse”* (Sri Lanka).

Some participants reported acceptance and positive attitudes from city and educated people helps RTO (six countries).

*In our society, in the urban area, people don't face these types of challenges, from the community, but in the village area they have some difficulties”* (Bangladesh).

Domestic access and support for activities of daily living (ADL), the first step toward RTO Was a challenge particularly in rural areas (five countries). While some modifications to improve mobility and ADL are available (seven countries), this represents a significant service gap (seven countries).

“*People don't get care for accessibility within houses, which make it difficult for them to take care of children and other activities they have”* (Afghanistan).

“*Transport and terrain access*” factors relate to roads, transport, infrastructure, or environment impact on RTO. Roads and transport systems/availability (10 countries), mountainous terrain (10 countries) and environmental factors e.g., heat/rain/flood, impose major challenges (six countries).

“*Some part of the country you can't go by car, you need to walk. Sometimes it is muddy and there are mountains that you need to carry your motorbike up and down which make it difficult for them to move”* (Cambodia).

“*Conflict and security issues*” in some countries impact the economy (Afghanistan/ Myanmar), rehabilitation, work availability and RTO (6 countries), and increase the numbers of LLA requiring RTO services (3 countries).

“*Afghanistan has been facing 40 years of war and as a consequence increasing the number of disabled”* (Afghanistan).

‘*Policy*' challenges to RTO include no or weak regulatory supports (seven countries), exclusion of rehabilitation/RTO services from national health systems (four countries), poor policy application (six countries) and complex bureaucratic systems to access services (five countries).

‘*Country service models*' impact individual RTO potential with inadequate government engagement (eight countries) and rehabilitation and RTO services being considered charity (four countries), and often provided for only short durations.

“*Pension scheme, vocational training center run by government. It's like a charity way, you come and do something. Then after that nobody know what happens. But it is not done systematically”* (India).

Consideration of local context is described in “*targeted service provision*,” where limited understanding of the population characteristics undermines the focus of RTO (five countries).

“*Nobody does that research to explore what should be done for these for the people to go back to work”* (India).

## 4. Discussion

### 4.1. Key findings

This study aimed to identify barriers to and enablers for RTO for people with LLA in East, South, and Southeast Asian developing countries, from the perspective of rehabilitation professionals. The views of the rehabilitation professionals provide a rich and unique data set, describing a largely different LLA population to that commonly found in many western countries (7). Despite their varied cultural, religious and geographical characteristics, consistent factors impacting RTO were identified among these thirteen countries (7, 28). While many of the population characteristics and impacting factors are similar to those found in LLA rehabilitation and RTO in developed countries, important differences include particular cultural and resource challenges, and trauma rather than disease as the most common cause of LLA in these countries (29).

Older people, females, the less educated, those with complex health issues, lacking services awareness, engaged in physically demanding work pre-LLA, and/or being from lower socio-economic groups, face greater challenges for RTO. Previous studies in other Asian and developed countries have also identified these challenges, suggesting the need for customized supports (4, 28, 30). Socio-cultural, economic and geographic factors further compound these challenges to RTO (1, 7, 31), including gender related issues frequently arising from religious beliefs which impact rehabilitation and RTO, particularly for those of Muslim faith. These issues have also been previously identified in rehabilitation management in developed countries, including challenges related to stigma and disability, cross gender interactions, and family priorities, often particularly disadvantaging females. Conversely, the prevalence of collectivist cultures in these Asian regions potentially provides opportunities for family and community involvement for implementation of RTO interventions including through CBR (22). These and other “*environmental*” factors substantially impact service provision at all levels and underline the importance of government initiatives including supportive regulation to improve RTO opportunities for people with LLA in this region (1, 7, 32, 33).

A biopsychosocial approach to rehabilitation providing continuity of care from acute management to community reintegration has been identified as essential for outcomes as close as possible to that pre-amputation (22). RTO includes return to self-care, productivity, leisure activities and community. The focus on physical services related to prosthetic provision and mobility neglects other essential aspects of management including mental health support and other reintegration activities important to improve RTO outcomes (34). Being able to manage personal hygiene and other everyday functions within domestic environments, and participation in community and vocational activities is essential (22).

Previous studies in Asian countries have found a lack of peer/family support, timely referral, ideal amputations and gender balance in rehabilitation teams (35, 36). The RTO stage of the LLA rehabilitation journey is primarily the responsibility of allied health professionals including physiotherapists, PO, occupational therapists, social workers and psychologists working in MDTs with rehabilitation clinicians where available (22). An important demographic difference in this region compared to other LLA populations is the youth of many of those affected who frequently have families reliant on them for income, further underlining the importance of reintegration (7). Collaborative rehabilitation management using MDT with RTO considerations from pre-amputation onwards, with maintenance of continuous care and involvement of all the relevant professionals throughout, has proven to be effective in developed countries (37). As well as recruiting sufficient numbers of appropriately skilled professionals, regulation of rehabilitation professionals' practice to promote quality care from acute-care to community reintegration (38) are particularly relevant needs for this region (7, 39). The interacting and interlinked challenges to RTO identified at all levels of the system support the need for mitigation strategies toward service delivery improvements informed by all stakeholders and implemented using systems approaches (28, 40, 41).

While opportunities exist to improve regional RTO services through diversifying health care models (42), resource availability (7, 43) and geo-political conditions (44), local customization of services is required (12, 32, 45). Additionally, the findings suggest the need for consideration of efficacy of western and/or charitable models utilized by NGOs (46, 47) without provision of sustainable support systems. Similar to reports from our study participants, Olavides-Soriano et al. (48) describe the challenges of RTO service provision in the Philippines, one of the targeted regional developing countries from which we were not able to recruit for this study (48). Barriers to service provision, implementation, and monitoring in low-resourced environments include multiple, overlapping and fragmented regulatory supports and government systems. In addition to legislative supports being implemented and enforced, family, community and workplace involvement has been identified as essential for RTO regardless of the level of development of the country with CBR and vocational centers providing models to potentially address implementation of inclusive disability initiatives (49). CBR has been used to both mitigate barriers to participation in all aspects of life and provide basic rehabilitation services at community level, including in the Philippines (50). Magallona1 and Datangel (2011) describe a model initiated by rehabilitation academics to develop contextually implemented sustainable rehabilitation solutions with outcomes including “*the change in viewing persons with disabilities as “persons” instead of “patients,” and the change in roles of persons with disabilities and their families from “recipients” to “active participants*” (p. 58) suggesting CBR as a powerful and adaptable tool toward improving reintegration services in low resourced environments (51).

### 4.2. Recommendations for future research

This study identified many factors, both barriers and enablers, interacting within the different layers of healthcare systems. Although they identified detailed and diverse factors impacting RTO, many of the participants acknowledged gaps in their understanding of RTO services and related policy level factors, suggesting the need for further research involving organizational and policy level stakeholders as well as more participants from more countries. Further research into the efficacy of the innovative services which are being provided as potential models suitable for other countries in the region, is also recommended.

### 4.3. Study strengths and limitations

Although qualitative research methods have some limitations, they provide an understanding of the complexity and background of actions to a particular problem (52). This study aimed to recruit participants from 25 countries but despite extensive measures, recruited from only 13. The use of a convenience sample may limit generalisability, and the results can only be those of the participant sample which may limit representation of experience in each country, and particularly in countries with only one participant (53). Some countries such as China have been identified as having policies limiting research participation (54). Interviewing online in English was a further potential barrier. The research was undertaken during the COVID-19 pandemic which may have impacted services including rehabilitation and created further difficulties reaching potential participants. However, data saturation was reached.

Regardless, this study provides new and rich information to inform a significant gap in the evidence base, toward understanding rehabilitation and RTO for people with LLA in this region.

## 5. Conclusion

Interlinked key factors were identified at different levels of healthcare service systems in developing Asian countries which impact RTO for people with LLA. This complexity reinforces the importance of systems approaches to best utilize limited resources toward improving RTO service delivery in this region. Identifying common factors impacting RTO across these countries also emphasizes the need for rehabilitation services to address occupational restoration as well as mobility. The adoption of sustainable and customized rehabilitation strategies is important, with multi-stakeholder participation based on individual patient needs. Finally, comprehensive approaches addressing all levels of healthcare systems and targeting reintegration of people with LLA back into their pre-LLA life should be embedded in and supported by sustainable and appropriately resourced policies throughout this region.

## Data availability statement

The raw data supporting the conclusions of this article will be made available by the authors, without undue reservation.

## Ethics statement

The studies involving human participants were reviewed and approved by this study was performed in line with the principles of the Declaration of Helsinki. Approval was granted by the Ethics Committee of La Trobe University (Date: 19 August 2021/No. HEC21196). The patients/participants provided their written informed consent to participate in this study.

## Author contributions

Material preparation, data collection, and analysis were performed by MS, JO, and RS. The first draft of the manuscript was written by MS. All authors commented on previous versions of the manuscript, contributed to the study conception and design, read, and approved the final manuscript.
