# Supplementary material for: Rehabilitation professionals' perspectives of factors influencing return to occupation for people with lower limb amputation in East, South, and Southeast Asian developing countries: A qualitative study
Source: Front Public Health. 2023 Mar 2;11:1039279. doi: 10.3389/fpubh.2023.1039279 (PMC10018026; doi:10.3389/fpubh.2023.1039279)
Supplement: Supplementary file 1 [file Table_1.DOCX]

**Guided Interview Questions**

***Part A: Preliminary Demographic questions (to understand the participant):***

1. Tell me about you and your involvement with the rehabilitation of the people with LLA.

Prompts: age/ gender/ profession/ institution or place of professional education/ time in profession/ time involved with rehabilitation for people with LLA/ where you work / how busy your rehabilitation centre is?

***Part B: Rehabilitation profile (background information):***

1. *Tell me about your country’s rehabilitation process and how people with amputation are managed?*

*Prompts: service availability/ government involvement/ rehabilitation professionals’ involvement/ professional regulation/ cost of rehabilitation/ financial support for rehabilitation/service costs/ referral, access timing after amputation/barriers and enablers for access to and participation in rehabilitation*

1. *Tell me about re-integration of people with LLA back into their life after rehabilitation?*

*Prompts: supports for rehabilitation and return to work - government and other national and international organizations/ availability of support - everyone or sectors / effectiveness - is helping people to return to work of their previous status - how*

1. *Tell me about the strategies used to involve people in need?*

*Prompts: strategies for all people - specific groups/ links with acute care settings/ government provided rehabilitation services/ funding sources/diversity or responsive language or culture acknowledgment or strategies/ gender issues strategies/ peer support*

1. *Tell me about the professionals involved in rehabilitation for people with LLA in your country?*

*Prompts: roles/ gender balance/ teamwork support between professions?*

1. *Tell me about rehabilitation services (follow-up) when people with LLA return to community after finishing their first rehabilitation?*

*Prompts: follow-up arrangements/ cost of follow-ups/ support for follow-ups/ local prosthetic repairability/ accommodation for visits/ waiting lists for follow-up*

1. *Tell me about your experience with people with LLA returning to work after rehabilitation?*

*Prompts: availability and type of work/ new work existing work/ community attitudes and supports/ barriers and enablers*

***Part C: Recommendations for improvement:***

1. *What do you think could assist to improve access to and participation in rehabilitation for people with amputation?*
2. *What do you think could assist to improve return to work after rehabilitation for people with amputation?*
